# Analysing the natural population growth of a large marine mammal after a depletive harvest

**DOI:** 10.1038/s41598-017-05577-6

**Published:** 2017-07-13

**Authors:** M. A. Romero, M. F. Grandi, M. Koen-Alonso, G. Svendsen, M. Ocampo Reinaldo, N. A. García, S. L. Dans, R. González, E. A. Crespo

**Affiliations:** 1 Instituto de Biología Marina y Pesquera Almirante Storni, Escuela Superior de Ciencias Marinas - Universidad Nacional del Comahue, San Martín 247, 8520 San Antonio, Oeste (RN) Argentina; 2 Consejo Nacional de Investigaciones Científicas y Técnicas (CONICET), Buenos Aires, Argentina; 3 Laboratorio de Mamíferos Marinos, Centro para el Estudio de Sistemas Marinos (CESIMAR) CCT-CENPAT-CONICET, Bvd. Brown 2915, 9120 Puerto Madryn, Chubut Argentina; 4 Northwest Atlantic Fisheries Centre, Fisheries and Oceans Canada, 80 East White Hills Road, St. John’s, A1C 5X1 Newfoundland and Labrador Canada; 5 Universidad Nacional de la Patagonia San Juan Bosco, Bvd. Brown 3051, 9120 Puerto Madryn, Chubut Argentina

## Abstract

An understanding of the underlying processes and comprehensive history of population growth after a harvest-driven depletion is necessary when assessing the long-term effectiveness of management and conservation strategies. The South American sea lion (SASL), *Otaria flavescens*, is the most conspicuous marine mammal along the South American coasts, where it has been heavily exploited. As a consequence of this exploitation, many of its populations were decimated during the early 20th century but currently show a clear recovery. The aim of this study was to assess SASL population recovery by applying a Bayesian state-space modelling framework. We were particularly interested in understanding how the population responds at low densities, how human-induced mortality interplays with natural mechanisms, and how density-dependence may regulate population growth. The observed population trajectory of SASL shows a non-linear relationship with density, recovering with a maximum increase rate of 0.055. However, 50 years after hunting cessation, the population still represents only 40% of its pre-exploitation abundance. Considering that the SASL population in this region represents approximately 72% of the species abundance within the Atlantic Ocean, the present analysis provides insights into the potential mechanisms regulating the dynamics of SASL populations across the global distributional range of the species.

## Introduction

Over thousands of years, humans have exploited animal populations by fishing, hunting or harvesting, causing long-term changes in population abundances. At the same time, human activities have impacted the underlying natural dynamics of many populations through habitat degradation and biodiversity loss^1^. These effects at the population level are then connected with changes to community structure and functioning that affect the provision of fundamental ecosystem services^2^. In this context, the prediction population dynamics is one of the mainstays of theoretical and applied ecology.

Models of population growth have many implications for management and conservation. Such models have been used to estimate extinction risk^3, 4^, harvest rates for exploited populations^5, 6^ and to predict the potential recovery rates of depressed populations^7, 8^. Models of exploited populations are used to understand the interplay of additional sources of mortality, such as human-induced, with natural mechanisms^9^.

The South American sea lion (SASL, *Otaria flavescens*) is the most conspicuous marine mammal along the South American coasts, where it has been heavily exploited. As a consequence of this history of exploitation, many of its populations were decimated during the early 20th century^10, 11^. Sea lions were taken mainly for leather and oil. Even though the harvest ceased by the 1990s, SASL populations are still exposed to several potential negative impacts linked to the development of marine coastal human activities (see 12 for a review). Today, the global SASL abundance is estimated to be approximately 400,000 animals, but local population trends vary widely within its range^12^. The populations in Uruguay and southernmost Chile are decreasing^13, 14^, those in central and southern Chile are stable^15, 16^, while the populations in the Falkland (Malvinas) Islands, Peru, and northern Chile are slowly recovering from very low levels^17, 18^.

In Argentina, the most intense harvest occurred from 1920 to 1950 in northern Patagonia and Tierra del Fuego^19, 20^, impacting two distinct demographic units^12, 21^. The northern and central Patagonia population is steadily recovering, growing at an annual rate of 5.7%^22, 23^. However, current fisheries and coastal development could impact the rebuilding capacity of this population^12^, and it is expected that risk analyses will be needed to properly manage this population in the future. One important concern in such analysis is the population dynamics, specifically how the population responds at low densities. At present, few attempts have been made to model the dynamics of any South American sea lion population.

The robustness of the predictions of population models, when used in the management and conservation of marine mammals, is usually limited because data are scarce and discontinuous^24^. However, the population of *O*. *flavescens* along the northern and central Patagonian coast of Argentina has been effectively monitored since 1972. This monitoring has produced an unusually long and detailed time series for the study of marine mammal population dynamics. Additionally, historical data about sea lion exploitation, together with coarse estimations of population abundance from the late 1930s and 1940s are also available^19, 20^. Based on these data, it has been found that the northern and central Patagonia population dropped drastically in a few years (<10% of their pre-exploitation values). Even though the harvest ceased in 1962, the recovery of the population only became apparent in the early 1990s after 3 decades with no detectable change in population size^25^. When all these elements are considered, it becomes evident that the Patagonian SASL population provides a unique opportunity to investigate a natural experiment on reduction and posterior recovery in a marine mammal species.

**Figure 1:**
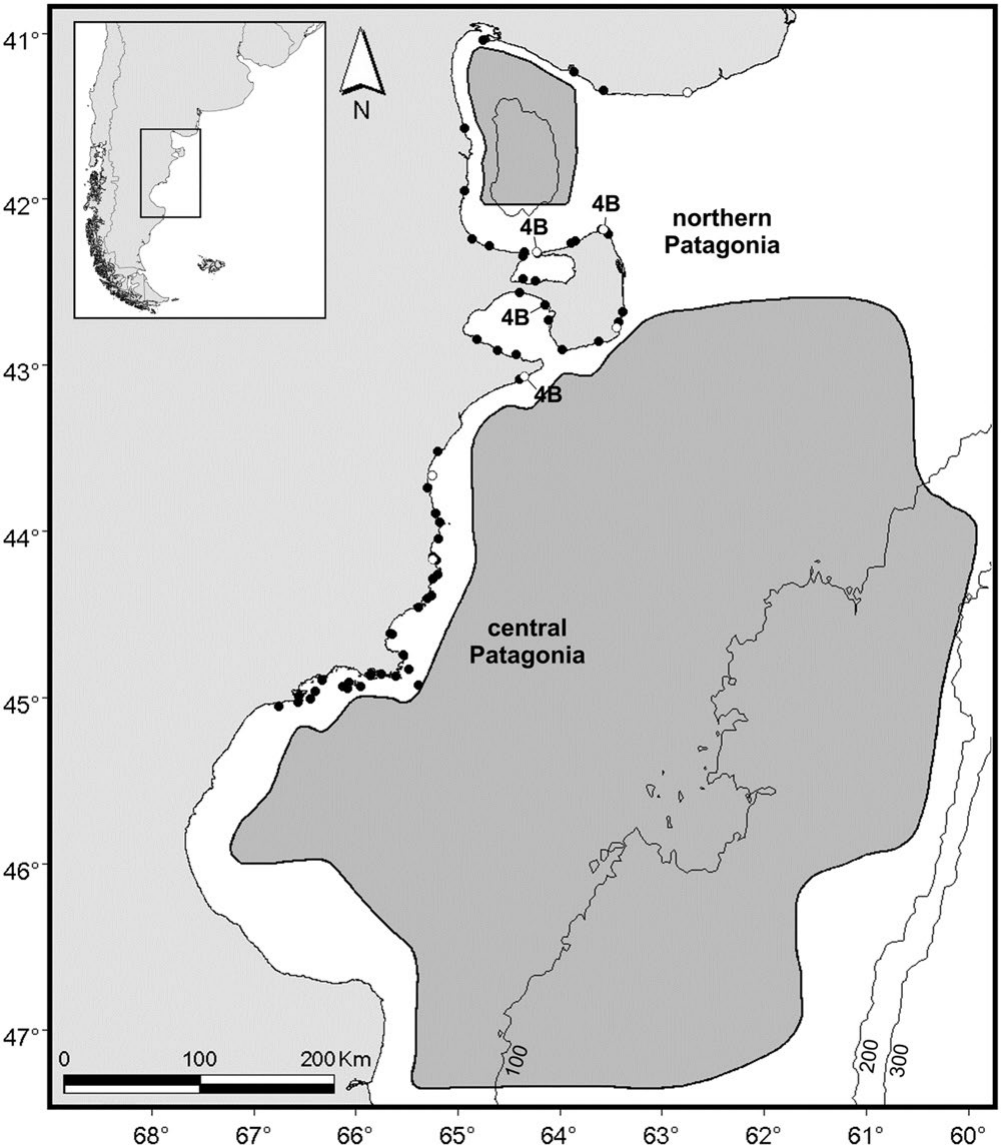
Detailed studied area at Patagonia Argentina with the current distribution of *Otaria flavescens* colonies. ○: rookeries commercially exploited, ●: other colonies, 4B: four key and historically important breeding colonies. Grey polygons indicate area of operations of the trawling fishery in northern and central Patagonia, where bycatch rate were obtained. Map generated with ArcGIS 9.3, (http://www.esri.com/software/arcgis).

We approached this exploration by applying a state-space model (SSM) framework to assess *O*. *flavescens* population recovery after a depletive harvest. From an ecological perspective, we were particularly interested in understanding how the population responds at low densities, how human-induced mortality interplays with natural mechanisms, and how density-dependence may regulate population growth. To achieve this, we summarized the sea lion harvest and bycatch history for the northern and central Patagonia population during the last century and integrated this information with recent abundance estimates using Bayesian state-space dynamic models. Understanding the population dynamics of this population, particularly the patterns and processes involved in the recovery, is essential to properly managing sea lions in the northern and central Patagonia region (Fig. 1). The results from this study can also provide insights into the potential mechanisms regulating the dynamics of other SASL populations, and thus contribute to understanding the observed differences in population trends across the global distributional range of the species. Overall, these results are expected to contribute to broader conservation efforts for SASL populations and constitute a baseline for future multispecies modelling studies about the effect of sea lion population recovery on the marine ecosystem in northern and central Patagonia, Argentina.

## Results

The estimated annual SASL harvest rates for the northern and central Patagonia populations ranged between 7 and 17,907 animals per year, reaching their maxima between 1938 and 1941 (see Supplementary Table S1). Sea lion bycatch occurred in all types of trawl fishing. Capture rates ranged from 0.002 to 0.02 sea lions per boat per day, with the highest rates being estimated for daytime bottom trawls directed at hake and pelagic nocturnal trawls targeting shrimp^26, 27^. The total annual mortality throughout the time series ranged from 216 to 1,703 animals per year, depending on the bycatch assumptions considered (see Supplementary Table S1). Compared to commercial harvesting, bycatch levels remained relatively low since the very beginning of the fishery and had no impact on the model estimates (i.e., model runs with and without bycatch had similar overall performances).

**Figure 2:**
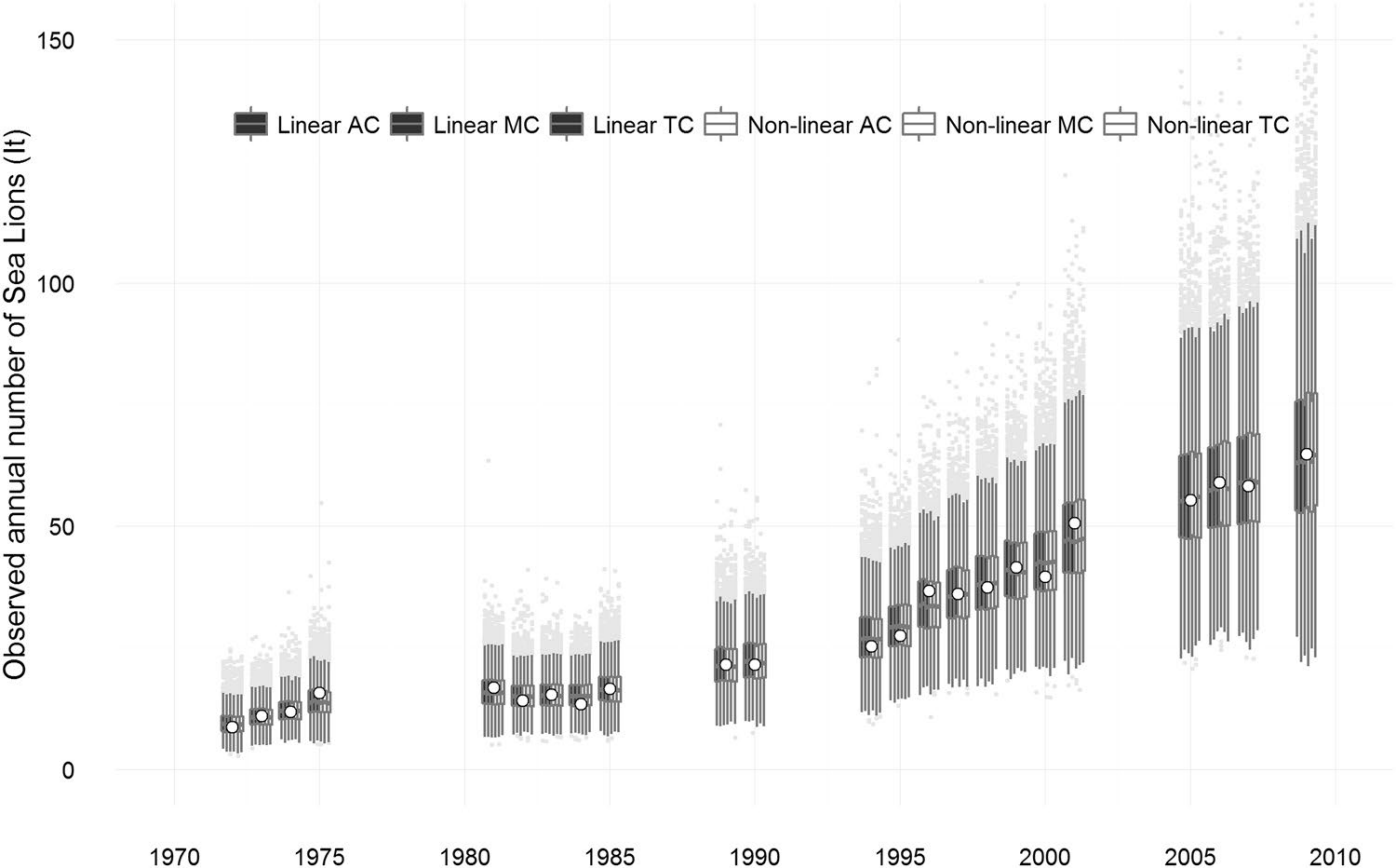
Trend of observed annual number of South American sea lion *Otaria flavescens*. Time series of observed annual number (white dots), together with posterior medians (horizontal dashes in boxes), first and third quartiles (boxes), and 2.5/97.5 percentiles (whiskers), provided by (from left to right) the linear and non-linear density-dependence models (*I*_*t*_) according to the three bycatch estimated series. TC: Total Catch set; AC: Average Catch set; MC: Maximum Catch set.

Overall, the implemented models performed well and provided good estimates of population levels while predicting reasonable dynamics for the SASL population from northern and central Patagonia. Input data points (I_t_) were compared with the median and 95% credible intervals of the corresponding posterior predictive distriutions for all proposed models. Nearly all observed abundance points fell within the 25^th^ and 75^th^ percentiles of the predictions provided by the six models considered (linear and non-linear density-dependence models under the three different bycatch series) (Fig. 2). Even though all models provided satisfactory fits under both the linear and non-linear density-dependence scenarios, the non-linear density-dependence models had slightly better predictive performances that reduced the uncertainty in the model outputs, hence producing smaller credible intervals.

Summary statistics for all posterior distributions are presented in Table 1. In general, posterior estimates of the maximum rate of increase (R_max_), detectability coefficient (*q*), process variance (σ^2^) and observation variance (τ^2^) remained relatively constant across different model formulations, but the non-linear density-dependence models had slightly less uncertainty for all parameters (i.e., narrower 95% Bayesian credible intervals). Means were generally greater than medians, showing positive skewness. The posterior median for R_max_ was 0.06 for the linear density-dependence models and 0.055 for the non-linear density-dependence models. The observation error was slightly greater than the process error. The standard deviation of σ^2^ was estimated to be almost identical among the six proposed models, with posterior medians between 0.084 and 0.086. The observation variance *τ* ^2^ showed similar trends, with a posterior median of 0.098. Posterior distributions for *q* had medians between 0.53 and 0.57. In the non-linear density-dependence models, the posterior medians for the shape parameter (*z*) were all significantly >1 (Table 1), varying between 5.8 and 6.12. This indicated a left-skewed surplus-production function (when *z* > 1, the surplus production is higher when abundance is above ½ of *K*) for the sea lion population. The main difference between the two alternative structural model formulations was the estimated pre-harvested population abundance, which was set at the environmental carrying capacity (i.e., *N*_1900–1928_ = *K*). The estimated *K* for the linear density-dependence models was approximately 25% lower than that in the non-linear density-dependence models (Table 1). In all cases, the posterior CV for *K* was lower (i.e., ranged from 73% to 82%) than the value used in the prior CV (100%). The marginal posterior densities for all parameters are shown in Supplementary Figure S1, together with their respective prior densities. Despite the relatively wide posterior distributions, most distributions were updated to lower or higher values, suggesting that survey data were informative for parameter estimation. Correlations among posterior parameter distributions were low for all models (see Supplementary Table S2), allowing for the estimation of individual parameters.

**Table 1:** Parameter estimates (posterior mean, standard deviations and credibility intervals) for maximum rate of increase (R_max_), carrying capacity (*K*, expressed in thousands of individuals), detectability coefficient (*q*), process variance (σ^2^), observation variance (τ^2^) and shape parameter (*z*) derived from the six Bayesian state-space surplus production models (linear and non-linear density-dependence models under the three bycatch indices).

Model	Parameter	Mean	St. dev.	Bayesian credibility intervals		
				2.5%	Median	97.5%
Linear density-dependence						
TC	R_max_	0.070	0.044	0.016	0.06	0.184
	*K*	294.3	216.3	75.4	235.7	857.6
	*q*	0.535	0.222	0.118	0.542	0.919
	*σ* ^2^	0.093	0.038	0.043	0.085	0.189
	*τ* ^2^	0.105	0.036	0.055	0.098	0.194
AC	R_max_	0.069	0.044	0.016	0.059	0.183
	*K*	299.9	224.9	78.3	239.5	888.7
	*q*	0.532	0.224	0.111	0.542	0.918
	*σ* ^2^	0.092	0.038	0.043	0.085	0.188
	*τ* ^2^	0.105	0.036	0.055	0.098	0.193
MC	R_max_	0.073	0.046	0.016	0.062	0.191
	*K*	297.3	223.5	77.4	238.7	867.4
	*q*	0.526	0.221	0.117	0.532	0.915
	*σ* ^2^	0.094	0.039	0.044	0.086	0.190
	*τ* ^2^	0.105	0.036	0.055	0.098	0.194
Non-linear density-dependence						
TC	R_max_	0.062	0.035	0.015	0.055	0.146
	*K*	420.6	343.1	97.1	319.3	1346
	*q*	0.558	0.21	0.156	0.566	0.922
	*σ* ^2^	0.091	0.036	0.043	0.084	0.182
	*τ* ^2^	0.104	0.036	0.055	0.098	0.193
	*z*	5.518	2.852	0.376	5.817	9.803
AC	R_max_	0.062	0.035	0.015	0.055	0.147
	*K*	427.5	354.6	96.5	324.3	1379
	*q*	0.563	0.21	0.158	0.572	0.923
	*σ* ^2^	0.093	0.038	0.044	0.085	0.187
	*τ* ^2^	0.105	0.036	0.055	0.098	0.195
	*z*	5.626	2.843	0.392	5.958	9.816
MC	R_max_	0.065	0.037	0.016	0.058	0.151
	*K*	433.3	357.3	100.3	329.6	1385
	*q*	0.547	0.208	0.155	0.553	0.916
	*σ* ^2^	0.093	0.037	0.044	0.085	0.186
	*τ* ^2^	0.105	0.036	0.055	0.098	0.195
	*z*	5.753	2.814	0.431	6.128	9.832

TC: Total Catch set; AC: Average Catch set; MC: Maximum Catch set.

The performances of the different model variants can be compared through differences in the Deviance Information Criterion (*DIC*) and Bayesian *p*-values (Table 2). The effective number of parameters (*pD*), which should be a positive quantity, could not be estimated reliably in WinBUGS for any model. The alternative estimator of model complexity^28^, $pV=\frac{1}{2}Var(\bar{D})$, where $\bar{D}$ is the posterior mean of the deviance, was considered as an estimate of the number of free parameters in the models. This estimate generally turns out to be remarkably robust and accurate^28^. Therefore, based on the $DIC=\bar{D}+pV$, the non-linear density-dependence models provided a slightly better fit to the data than the linear density-dependence models (Table 2). Among non-linear density-dependence models, the model considering the Total Catch (TC) index for the bycatch series showed the lowest value of *DIC*. Nevertheless, there was no meaningful difference between all six models based on the *DIC* since its range was <2. An inspection of the standardized residual plots also confirmed that there were no systematic deviations in any of the models (see Supplementary Fig. S2). The Bayesian posterior mean abundance and observed counts, when centred on a 1:1 line, indicated good fit of all models to the observed data. Posterior checking revealed no inconsistency between the model *a posteriori* and the data. All six models had predictive Bayesian *p*-values close to 0.7 (with 95% C.I.s that include 0.5), indicating good *a posteriori* ability of all models to replicate the abundance data.

**Table 2:** Model performance for six alternative models (linear and non-linear density-dependence models under the three bycatch indices) shown as Bayesian *p*-value, and *DIC* values giving the estimator of model complexity (*pV*).

	Bayesian *p*-values	*pV*	*DIC*
Linear density-dependence			
TC	0.70	29.35	179.80
AC	0.70	29.03	179.44
MC	0.71	29.00	179.47
Non-linear density-dependence			
TC	0.70	28.71	178.90
AC	0.70	28.90	179.02
MC	0.70	28.62	179.06

TC, AC, and MC respectively denote Total Catch, Average Catch and Maximum Catch set estimated to reconstruct bycatch history.

Convergence diagnostics were compiled to see if there were any problems with convergence in the MCMC simulations. Based on the Geweke test, the six models converged adequately in all three chains (i.e., *p* > 0.05). The Gelman and Rubin statistics for all parameters equalled 1.0, providing no evidence for a lack of convergence in the distribution of the MCMC samples with the posterior distribution ($\hat{R}$). All parameters also passed the two-stage Heidelberger-Welch stationary test. The autocorrelation function plot indicated that a thinning interval of 50 was large enough to address potential autocorrelation in the MCMC runs. Lastly, the trace plots showed a satisfactory mixing of the three chains. Overall, the convergence diagnostics indicated that all models can be considered to have reliable results, the posterior distributions of the model parameters having been adequately sampled by the MCMC simulations.

**Figure 3:**
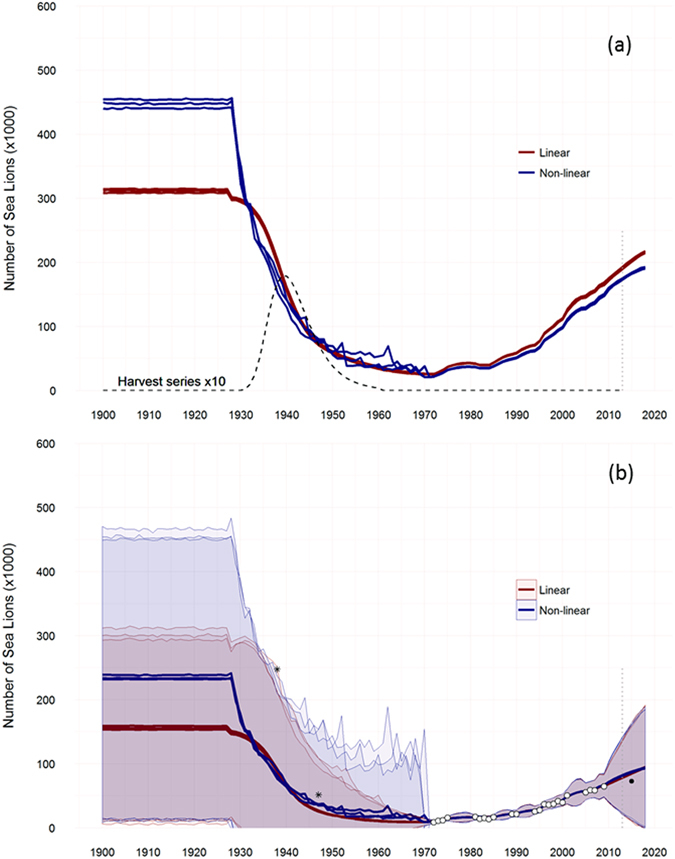
Abundance trajectories for all proposed models. (**a**) Posterior distribution of mean population abundances (*N*_*t*_) and harvest time-series (dashed line). (**b**) Predicted posterior mean (±SD) of observable states (*I*_*t*_) compared to the observed survey data (white dots). Asterisks represent the first two coarse estimates of population abundance of sea lions in Argentina^19, 20, 69^. Black dot corresponds to survey data for 2015. This data point was not included in the model fitting exercise.

The population trajectories (posterior distribution of mean abundances, *N*_*t*_) for all proposed models showed a similar trend, with a rapid and severe depletion of the SASL population by hunting and the lowest abundances levels appearing between 1950 and 1980 (Fig. 3a). By the early 1970s, the population was estimated to be at <10% of the pre-exploitation abundance for both types of model. Thereafter, the models estimated a recovery in terms of population abundance at a growth rate close to R_max_ despite the individuals taken by incidental catches. There are essentially no differences between the mean estimates of current population size between models. The non-linear density-dependence models estimated that the total population abundance in 2013 was between 172 and 175 thousand individuals compared with an abundance ranging from 189 to 192 thousand individuals estimated by the linear density-dependence models. The pristine population (here assumed to correspond to *K*) was generally less well estimated. The two models produced different population trajectories for the pre-1940 period. The trends for the predicted posterior means of observable states (*I*_*t*_) from the six models are shown in Fig. 3b and compared to the observed abundance and catch series. Both models underestimated the survey from 1938, but the posterior means of the observable states (*I*_*t*_) from the linear density-dependence models seemed far too low to be consistent with this survey. The early population estimate for 1947–1949 was relatively well approximated by all models, although these survey data may be underestimated.

Considering the sequential inference procedure, it is straightforward to estimate the one-step ahead density of the states (*N*_*t*+1_). The projected observable states (*I*_*t*_) of the SASL population for 2015 had posterior means ranging between 86.27 and 88.10 (SD: 73.14–76.26) thousand individuals in the non-linear density-dependence models and between 84.47 and 86.83 (SD: 73.09–77.33) thousand individuals in the linear density-dependence models (Fig. 3b). These values were much lower than the pre-exploitation abundance but higher than the observed data obtained from a terrestrial survey that year (72.59 thousand individuals) for northern and central Patagonia. This suggests a likely slow-down in population growth.

**Figure 4:**
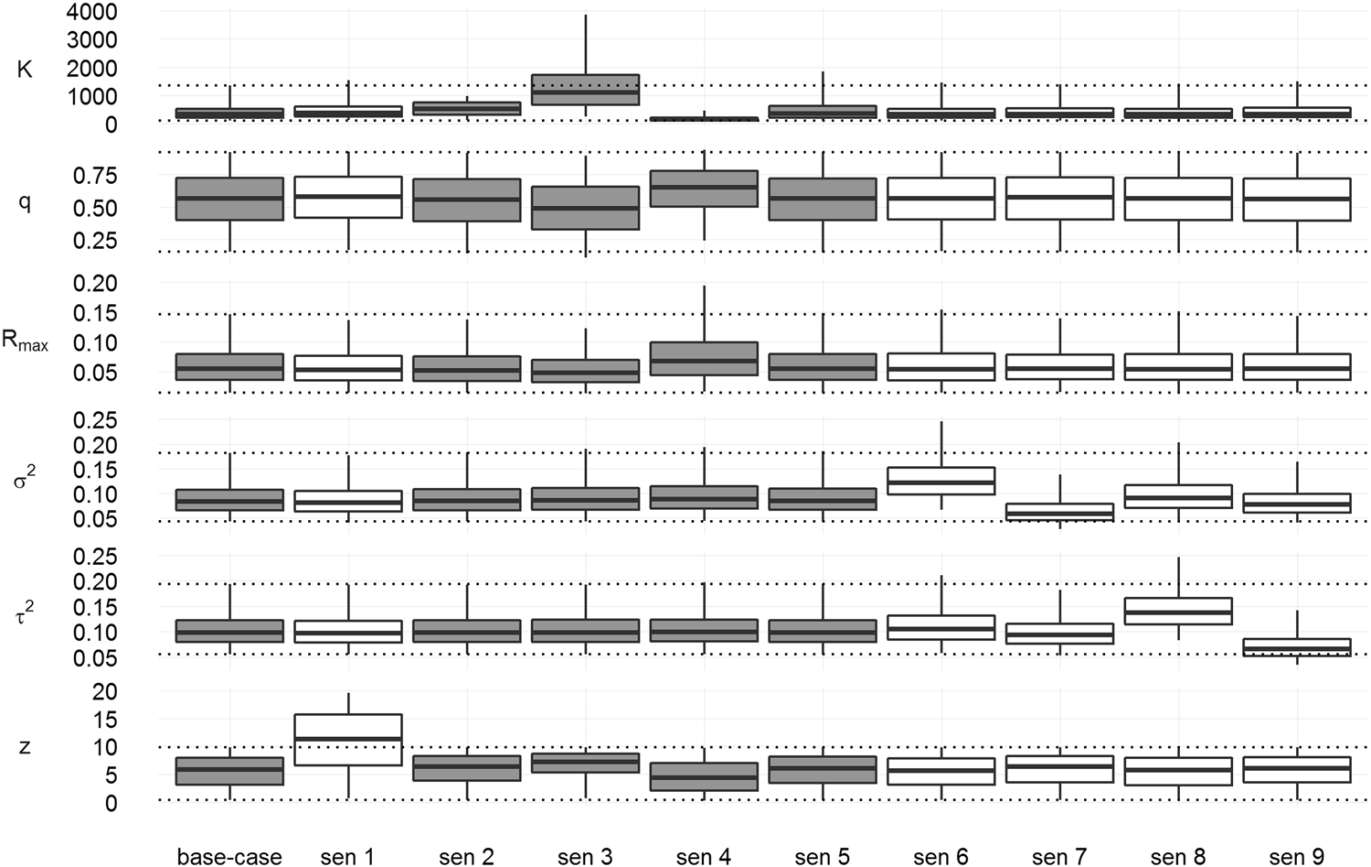
Sensitivity of model parameters to prior probability specifications. Median estimates (solid black lines in boxes), first and third quartiles (boxes), and their 95% C.I. (bars) are presented. For each parameter considered (detectability coefficient *q*, carrying capacity *K* (in thousands), maximum rate of increase *R*_*max*_, shape parameter *z*, process variance σ^2^ and observation variance τ^2^), two grey dotted lines indicate as references the 95% confidence intervals obtained with the base-case model. Sen 1 modified *z*, sen 2–5 modified *K* and sen 6–9 modified σ^2^ and τ^2^. See Table 5 for a description of sensitivity analyses.

A sensitivity analysis of parameter estimates to prior probability specifications was conducted considering the non-linear density-dependence model with the Total Catch (AC) series of bycatch estimates as the base-case model. Moreover, as the goodness-of-fit statistics did not provide clear advice on model selection, this model was chosen among all the candidate models based on its slightly lower *DIC*, lower uncertainty for all parameters and posterior predictive distributions of the data and lesser likelihood to underestimate the pre-exploitation population abundance. The examination of the model results considering plausible alternative variants of the base-case prior distributions showed that the data appeared to be informative for R_max_ and *q* because the marginal posterior distributions for these parameters were quite insensitive to different choices of priors (Fig. 4). The estimated *K* was sensitive to the choice of its own prior distribution, which suggests that the data were not strongly informative with respect to carrying capacity. Specifications with lower carrying capacities showed a slight increase in the maximum rate of increase and detectability coefficient and a decrease in the shape parameter. We had no strong evidence as to what the upper limit of the carrying capacity might be, but increasing the upper limit further did not seem to significantly influence the posteriors of the other parameters in the model. The posteriors for *z* and the process and observation error variance were also sensitive to their prior distribution, but the effect on the other estimated parameters was limited (Fig. 4).

## Discussion

We used Bayesian inference to integrate the time series of population abundances, commercial harvests, and fisheries bycatch into dynamic SSMs for the South American sea lion population from northern and central Patagonia. We relied on previous studies to define plausible prior distribution for the parameters and to set the likelihood function. This is particularly important for Bayesian inference because misspecification of prior distributions and the choice of an inappropriate likelihood function may result in unreliable posterior distributions for parameters^29, 30^. In the absence of a priori knowledge, as is the case for *K*, *z*, *σ* ^2^ and *τ* ^2^, it was crucial to assess the sensitivity of credible intervals to the choice of distribution for the vaguely informative priors considered.

We should also consider the form of the process and observation equations, as well as the distribution of process noise, in order to assess the appropriateness of the proposed models. In this study, we represented the process dynamics using the theta-logistic equation, which has a shape parameter that allows for the exploration of different relationships in the representation of the density dependence. Because this model is relatively simple and easy to implement, it is feasible to use more than one alternative formulation of the production function and conduct hypothesis testing^31^. Unfortunately, there were no age-structured harvest or bycatch data available for the SASL population to explore more complex Bayesian age- or size-structure models. The observation equation assumed that the survey data were proportional to the relative abundance. Using survey data avoids several problems that have been noted regarding the use of catch rates as a relative abundance indices, which is a common practice in fishery models^32, 33^. On the other hand, as expected, missing values in the data lead to wider posterior credible intervals for the abundance estimates. The Bayesian framework allows us to quantify the increased uncertainty for missing values and to potentially incorporate information from additional sources via the corresponding prior distribution. The process noise can come from several sources, mainly from demographic variability and/or environmental variability^34, 35^. We assumed a multiplicative lognormal error structure considering that the process noise represents the joint effects of a large number of random multiplicative events that combine to form a multiplicative lognormal process error. We also considered multiplicative structure for observation error, which seems to be the most appropriate structure for biological or ecological models^33^. The observation error was found to be slightly greater than the process error, irrespective of the model used (Table 1). It is not surprising that a long-lived, slow-growing and late-maturing species such as the SASL would display few temporal fluctuations in the aggregated population abundance reflected in the slightly low process error.

The Bayesian SSM framework implemented here can be considered an efficient tool to assess the population recovery of the SASL in northern and central Patagonia. The low correlation between process and observation variances and the relatively similar magnitudes of these parameters indicate the absence of any major estimation problems, which have been recently associated with Gaussian SSMs^35, 36^. We found that the data on SASLs provided enough information to estimate all parameters in these six models, although there were some differences in the posterior distributions of some parameters (for example, carrying capacity, *K*) and abundance estimates. After an examination of all model results, the non-linear density-dependence model with the Total Catch (TC) bycatch series was selected as the most plausible model (i.e., narrower 95% Bayesian credible intervals for parameter and abundance estimates). This indicates that the observed population dynamics of SASLs have a non-linear relationship with density, assuming an “overcrowding” or compensatory density-dependent process that affects the population growth rate at high densities. The non-linear relationship adds flexibility in the shape of the surplus production function to model the population growth behaviour^37^, and this property leads to relative higher estimates of the carrying capacity. From a conservation perspective, the selected model resulted in a higher carrying capacity, higher depletion level and lower recovery than that those estimated by the linear density-dependence models. These results highlight the importance of conservation efforts for this species.

The model estimated that the maximum depletion level occurred towards the end of the 1960s, reducing the population to just under 25,000 individuals. Previous studies also suggest that after commercial harvesting, the abundance of SASLs from Patagonia was less than 10% of the original size^10, 22, 23^. Different hypotheses have been proposed to explain the decline in Atlantic populations of *O*. *flavescens*. Gerber and Hilborn^38^ indicate that the harvest levels of *O*. *flavescens* between 1930 and 1970 alone would not account for the observed declines of Falkland (Malvinas) Islands and Península Valdés populations. They suggested that impacts from fisheries and/or the oil industry may have contributed to the declines in these Atlantic populations. However, significant fishing activities in the Patagonian region started more than a decade after harvest activities ceased^39^. Additionally, off-shore oil and gas exploration is only just now commencing^40^. Therefore, proposing that the concurrent/cumulative effects of all these activities are responsible for the declines in the northern and central Patagonia population is not a valid hypothesis. Other studies have suggested that SASL colonies in Argentina were unable to sustain the reported level of exploitation, thereby necessitating a migration from the Falkland (Malvinas) Islands to account for the number of SASLs killed^17, 41^. Recently, Baylis *et al*.^8^, using census data, and Hoffman *et al*.^42^, using molecular markers, tested this hypothesis and found that there is no inherent reason to assume that a winter migration from the Falkland (Malvinas) Islands was necessary to account for the number of SASLs killed in Argentina. Furthermore, our analysis shows that the addition of a simple population reduction variable, such as the available harvest data, successfully explains both the abrupt decline and the subsequent trend of the population abundance in the study area (Fig. 3).

Estimating the pre-exploitation abundance of marine mammal populations is not simply of historical interest; it is essential to understanding the true impact of exploitation on the marine ecosystem and has important management implications for the rebuilding and conservation of these populations^43, 44^. The most plausible model obtained in this study suggested that the pre-harvest SASL population abundance in northern and central Patagonia was approximately 420,000 individuals. The weaker performance in estimating this parameter was associated with the lack of good quality data on historical abundance. On the other hand, the generalized logistic model used assumes that carrying capacity is constant through time. The carrying capacity of a marine mammal such as *O*. *flavescens* can be defined as a function of both food availability and space to reproduce. The reduction in abundance suffered by SASL over the past century was followed by a decrease in its primary prey, Argentine hake *Merluccius hubbsi*, due to the large impact of high-seas fisheries^45–47^. These changes led to a severe reorganization of the whole ecosystem off northern and central Patagonia^46, 48, 49^. All these changes may suggest that the carrying capacity for SASLs could have changed over time and is now smaller than the value of *K* (the pre-exploitation abundance) estimated by the model. This may explain the slow-down in population growth that was observed when comparing the last survey with the projected observable states. Additionally, a recent study on craniometrical variables found that the somatic growth of SASL is density-dependent and suggested that industrial fishing has reduced the SASL carrying capacity of the ecosystem off Patagonia^49^. Similarly, the carrying SASL capacity in the San Matías Gulf seem to be constrained by the fishing fleets that have been removing and replacing several fish predators in the food web since 1971, affecting at the same time the prey of SASL^50^.

The Antarctic fur seal *Arctocephalus gazella* population in the South Shetland Archipelago currently has a trajectory converging on a tightly bound oscillation around an apparent equilibrium (the current carrying capacity), which is an order of magnitude lower than those levels before exploitation^51^. Huke-Gaete *et al*.^51^ suggest that this stable equilibrium may slowly be reached through a saltatory pattern in the next 100 years or so or that the suspected pre-exploitation levels are unlikely to be attained again as a consequence of major changes to the Southern Ocean, and as such, the population will remain within the current levels, resulting in an alternative stable state^51^. Currently, the recovered SASL population still represents only 40% (175,000 individuals) of the pre-exploitation abundance. In this context, it would be necessary to test whether there is a new equilibrium state in the *O*. *flavescens* population trajectory with a new dynamic model that incorporates a function of *K* with time as well as other components of its community.

The last commercial removals of *O*. *flavescens* were officially recorded in Patagonia in 1960^20^, and marine mammal harvesting was prohibited by a National Decree in 1974^52^. However, signs of population recovery were not detected until 1990, after 3 decades of stagnation^10, 22^. The model properly captured this delay in the recovery suffered by the SASL population. This relatively slow increase was interpreted at that moment as a consequence of fishery development^10^. Nevertheless, it was demonstrated three decades later that sea lion population increase was not only a consequence of fishery development but rather the interplay of multiple factors that act through a density-dependent mechanism^48, 53^. Genetic studies showed that there was not a loss of genetic variability due to overexploitation, as genetic markers have revealed no population bottleneck within the SASL population from northern Patagonia^54^. The population’s slow recovery can also be attributed to changes in environmental conditions (e.g., carrying capacity)^10, 22, 46^ combined with depensatory effects caused by reduced survival or fecundity. Considering that the survival of all age classes in northern Patagonia is higher in dense and big rookeries than in isolated or marginal breeding areas^53, 55, 56^, this vital rate could have had an effect on the regulation of the population growth rate.

After the initial absence of any measurable recovery, the SASL population started to grow, finally reaching a 5.7% annual rate of increase^22^. This observed rate, calculated through pup counts from 1983 to 2002, was similar to the *R*_*max*_ estimated here by the model. This fact suggests that after the depletive harvest, the population was increasing close to the maximum rate of increase, which is expected considering the nonlinearity in the density-dependence parameter. The resultant *R*_*max*_ also agrees with the suggestion that the reduction in the population rate of increase at low densities in otariid populations may not be strong^38^. The estimated maximum rate of increase for SASL populations is similar to those recorded for other recovering populations such as the California sea lion (0.052)^57^ and Steller sea lion in the Eastern Gulf of Alaska (0.059)^58^. However, this population has low rates of increase when compared with other otariids, such as *Arctocephalus* spp. with rates ranging from 0.04 to 0.26 for different species and populations^59–61^. Considering differences in body size and the age of first reproduction among sea lions and fur seals species^62^, this discrepancy would be expected but also highlights the importance of a knowledge on the biology of a species when it is necessary to use proxies from other species to parameterize dynamic models for management applications.

The South American sea lion faces several conservation problems related to the development of marine coastal human activities. Even though national and provincial laws in Argentina protect marine mammals, there is no framework that integrates the regulation of marine and coastal development with marine mammal conservation. This problem is particularly evident in relation to fisheries management regulations and is pervasive throughout most South American countries^63^. Currently, most international management rules employed for marine mammal populations need accurate estimates of population rates (like *R*_*max*_) and/or the environmental carrying capacity (*K*)^64^. Therefore, the estimated population parameters obtained here should be useful for monitoring the sustainability of current and future levels of incidental mortality and for any subsequent policies that could be designed to integrate the management of multiple activities at the marine ecosystem scale.

Simple single-species models can be very effective in evaluating specific scenarios, but we have to consider that populations are embedded in a complex web of species interactions, in which human activities could impact each component of the community in different ways. At present, the northern and central Patagonia population of *O*. *flavescens* is one of the more important reproductive nucleuses for the species and holds approximately 72% of the species abundance within the Atlantic Ocean^12^. Therefore, this analysis of its population growth after a harvest-driven depletion not only contributes to the understanding of the dynamic patterns and processes involved in its recovery but also provides a benchmark for comparison with other otariid populations. The baseline generated by this study should also contribute to the understanding required for developing an integrative ecosystem management plan for the northern and central Patagonia ecosystem.

## Methods

### Study area

The study area included the colonies located along the northern and central Patagonian coast, Argentina, between Punta Bermeja (41°09′S, 63°09′W) and Isla Quintano (45°14′S, 66°42′W) (Fig. 1). Genetic analyses based on molecular markers^54, 65, 66^, strong demographic connections, and similar population trajectories indicate a lack of geographical structure between these colonies^22, 25, 40^, which permits them to be treated as a single demographic unit.

### Annual harvest and bycatch estimates

The harvest activities occurred from 1929 to 1960 at seven rookeries within the study area (Fig. 1). Historical harvest data came from the number of leathers exported over five year periods by five factories from northern Patagonia^20^. In central Patagonia, commercial sealing occurred during a small number of isolated harvest periods at Isla Escondida in 1921 and at Punta Lobería in 1938–1940^20^ (Fig. 1). This area, in comparison with northern Patagonia, could almost be considered unexploited^25^. The information about leather exports^20^ is extremely useful because it represents the only record of the commercial exploitation of the SASL in Patagonia. Therefore, the annual harvest values (*H*_*t*_) were obtained as the difference between consecutive years from a cumulative catch curve (Gompertz equation with additive normal error) fitted to these available harvest data (see Supplementary Table S1).

Since the beginning of the 1980s, fisheries have developed in Patagonia using a diversity of methods ranging from trawls, jiggings, and longlines. Initially, eighty percent of the vessels operating in northern and central Patagonia were trawlers, but jigging efforts targeting squid have increased since the 1990s. The Patagonian trawl fleet is very heterogeneous and includes a diversity of vessels sizes, types of fishing and gear, targeting mostly white meat fish, which are important food resources for sea lions^45, 47^. This fleet has operated from 41° to 48°S (Fig. 1). The incidental capture of marine mammals by the Patagonian trawl fleet was recorded and quantified during 1992–1994^12, 26^. Taking into account the heterogeneity of the fleet, and assuming that net type could produce different effects on the marine ecosystem and different probabilities of marine mammal entanglement, the evaluation of the incidental mortality used nine fishing types defined according to trawl type, gear, target species, and time of operation (see the full fishing type description from 26,27). Among them, there were only seven recorded incidental catches of sea lions (see Supplementary Table S3). Bycatch rates were defined and estimated by Crespo *et al*.^26^ as the catch per unit of effort for each fishing type. Based on these bycatch rates and fishing effort time-series (from the Fisheries and Aquaculture National Department^67, 68^, see Supplementary Table S3), we reconstructed the SASL bycatch history for the period 1989–2013 under the assumption that the bycatch per unit effort rates remained constant during this period throughout the entire area. We postulated this assumption considering that fisheries did not show significant changes in fishing gear and location during this period (based on information available from the Fisheries and Aquaculture National Department). Conversely, recent estimates of bycatch per unit effort rates for the San Matías Gulf trawl fishery^12^ suggest that these rates remain at the same order of magnitude as those previously estimated by Crespo *et al*.^26^. Three time-series of annual bycatch values (*M*_*t*_) were estimated based on different assumptions about the potential level of impacts (see Supplementary Table S1):

1. Total Catch (TC) set:
  1a $${M}_{{t}_{TC}}=\sum _{i=1}^{n}TC{R}_{i}.{E}_{it}$$
  where *E*_*it*_ is the nominal fishing effort (number of fishing days per year) for each fishing type *i* (see Supplementary Table S3) and *TCR*_*i*_ is the Total catch rate for each fishing type (*TCR*_*i*_ values were taken from 26). This rate assumed that all vessels of each type exhibit the same bycatch rate per unit effort.
2. Average Catch (AC) set:
  1b $${M}_{{t}_{AC}}=\sum _{i=1}^{n}AC{R}_{i}.{E}_{it}$$
  where *ACR*_*i*_ is the average catch rate for each fishing type (*ACR*_*i*_ values were taken from 26). This rate assumed that there are variations in the catch between vessels under the same type, either by the vessel itself or by differences in the fishing methods of each crew.
3. Maximum Catch (MC) set:

1c $${M}_{{t}_{MC}}=\sum _{i=1}^{n}MC{R}_{i}.{E}_{it}$$

where *MCR*_*i*_ is the maximum catch rate for each fishing type (*MCR*_*i*_ values were taken from 26). This rate assumed a uniform catch rate that is equal to the maximum individual vessel rate within a fishing type. It assumed that each individual vessel exhibits the maximum bycatch rate per unit effort.

### Abundance estimates

The first two coarse estimates of SASL population abundance from Argentina correspond to the period of commercial exploitation^19, 20, 69^. Even though these historical data are invaluable, they were not used for model fitting because they were obtained with different methodologies. The estimate for 1938 was calculated from terrestrial surveys^69^ and could be over-estimated. The estimate for 1947–1949 was calculated from both aerial and terrestrial surveys^19^, but the sea lion abundance was probably underestimated because the reported figures correspond to the means of several surveys before, during and after the breeding season over different years. Although these earlier data cannot be used for model fitting, they still represent useful references for comparison with model results.

Abundance data were obtained from early surveys available from the literature and from our own surveys. The available dataset covered the period from 1972–2015 (Table 3, see Supplementary Table S1). For the periods 1972–1975 and 1981–1982, databases were available from Ximénez (1975)^70^, Castello *et al*.^71^, and Lewis and Ximénez (1983)^72^. These early survey data only included pup counts for the four main breeding rookeries (this set of four rookeries will be referred as 4B, Table 2, Fig. 1).

**Table 3:** Synoptic view of the available survey data of South American sea lion *Otaria flavescens* from northern and central Patagonia.

Year	Northern Patagonia		Central Patagonia	Correction factors applied
	Were all 4B rookeries surveyed?	Number of other sites surveyed	Was surveyed?	
1972	yes (only pups)	0	yes	all
1973	yes (only pups)	0	no	all
1974	yes (only pups)	0	no	all
1975	yes (only pups)	0	no	all
1981	yes (only pups)	0	no	all
1982	yes (only pups)	0	no	all
1983	yes	4	no	all
1984	yes	0	no	all
1985	yes	5	no	3
1986	no	1	no	—
1987	no	4	no	—
1988	no	1	no	—
1989	yes	3	yes	all
1990	yes	8	no	3
1993	no	3	no	—
1994	yes	6	no	all
1995	yes	10	yes	3
1996	yes	12	no	3
1997	yes	5	no	all
1998	yes	12	no	3
1999	yes	7	no	all
2000	yes	11	no	3
2001	yes	9	no	all
2002	no	7	no	—
2003	no	1	no	—
2004	no	1	no	—
2005	yes	18	yes	3
2006	yes	23	no	3
2007	yes	21	no	3
2009	yes	21	no	3
2010	no	3	no	—
2011	no	1	no	—
2013	no	5	no	—
2015	yes	21	no	3

4B are Punta Norte, Punta Buenos Aires, Punta Pirámide, and Punta León rookeries.

Annual direct counts of sea lions ashore were carried out during the period 1983–2015. Total counts were made coinciding with the peak of the breeding season (i.e., between the last week of January and 1^st^ week of February), when most of the individuals are present at the rookeries for reproduction and almost all of the pups are already born^52, 73^. The whole set of rookeries or haul-out sites were surveyed at the same time during the same breeding season whenever possible. However, in some years, both logistical and economic issues limited conducting surveys in certain subsets of colonies (Table 3).

To estimate population abundance, we developed three correction factors (given as the predicted values from a regression relationship) to be applied to the survey data (Tables 3 and 4). The first was used to estimate the non-pup fraction of the population in the periods 1972–1975 and 1981–1982. This factor was derived from the relationship between pups and non-pups in the 4B colonies (see Fig. 1) because these were surveyed in most years (Table 4). The second correction factor was developed to address the limitation emerging from incomplete survey coverage from 1983–2015 in northern Patagonia. During this period, not only were some surveys unable to cover all rookeries but the actual number of rookeries also increased, as new marginal breeding areas appeared as a result of population growth and changes in social structure^74^. To account for these effects, we developed a correction factor using the relationship between the total number of individuals counted in 4B rookeries and those counted in non-4B rookeries and haul-outs during the breeding season (Table 4). The third correction factor estimates the SASL abundance in northern and central Patagonia from a regression based on four surveys that considered both regions at the same time^23, 25, 75^ (Table 4). All these empirical relationships were applied to the survey data (Table 3) in order to estimate the total population abundance for 24 years between 1972 and 2015 (see Supplementary Table S1).

**Table 4:** Correction factors used to reconstruct the South American sea lion *Otaria flavescens* population abundance in northern and central Patagonia.

Dependent variable (*y*)	Independent variable (*x*)	Equation	*n*	*r* ^*2*^	Observations
Non-pups counted in 4B rookeries	Pups counted in 4B rookeries	*y* = 1.1152*x* ^*^	18	0.99	4B refers to four key and historically important breeding colonies**
Total number of individuals counted in non-4B rookeries and haul-outs in northern Patagonia	Total number of individuals counted in 4B rookeries	*ln(y)* = −19.982 + 3.1236*ln*(*x*)*	11	0.95	The data for a given year were included in this analysis, only if all 4B rookeries were counted and the other surveyed sites were considered to contain a large proportion of the remaining sea lion abundance at that time.
Abundance in northern and central Patagonia	Abundance in northern Patagonia	*y* = 1.815x*	4	0.99	This calculation assumes a fixed relative proportion between the sea lion abundance in northern and central Patagonia.

*Regressions were carefully examined, and their residuals evaluated to detect departures from the statistical assumptions.

**4B are Punta Norte, Punta Buenos Aires, Punta Pirámide, and Punta León rookeries.

### Model structure

The annual population abundance, harvest and bycatch estimates were integrated using a Bayesian state-space surplus production framework. This approach, originally developed for fisheries management, is regarded as powerful tool for modelling time-varying abundance indices because it simultaneously account for both stochastic process variability (the state model) and stochastic measurement error (the observation model)^35, 76–79^ and offers great flexibility in the mathematical construction of the model^77, 80^. SSMs have been increasingly applied in ecology to estimate the productivity and abundance of natural populations (e.g., refs 81, 82) since approaches that only account for one source of error can lead to biases in the model predictions and parameter estimates and may even mask the form of the underlying population dynamics, such as the degree of density-dependence^3, 83, 84^. Moreover, surplus production models may even occasionally provide better estimates of biological landmarks than age-structured models due to their high versatility and rather low parameter requirements^85^.

The model was run for the period 1900–2013. The survey data for 2015 were available but not used in model fitting; they were set aside for an *a posteriori* model performance evaluation. We predicted the size of the population in 2015 under the selected model based on the population, harvest, and bycatch information from previous years. The annual abundances between 1900 and 1971 were treated as unobserved random variables in the Bayesian modelling framework.

The basic population dynamic process was modelled using the following discrete formulation:

2 $${N}_{t}={N}_{t-1}+f({N}_{t-1})-{H}_{t-1}-{M}_{t-1}$$

where *N*_*t*_ is the unknown underlying state variable in year *t* (in this case, the unobserved annual abundance for the SASL population exposed to harvesting and bycatch, *t* = 1900, …, 2013), *H*_*t*_ is the number of animals removed by the commercial harvests in year *t*, *M*_*t*_ is the number of animals incidentally caught by the fishing fleet in year *t*, and $f(\cdot )$ is a surplus-production function. This function was specified as the following generalized theta-logistic equation^37, 86^:

3 $$f({N}_{t-1})={R}_{max}{N}_{t-1}[1-{(\frac{{N}_{t-1}}{K})}^{z}]$$

where *R*_*max*_ is the maximum rate of increase (i.e., the intrinsic population growth rate when *N*_*t*_ ~ 0), *K* is the carrying capacity and *z* is a shape parameter that controls the level of nonlinearity in the density-dependence. Two basic formulations were considered for density-dependence, one with *z* = 1 (i.e., linear density-dependence and gives the ordinary logistic, or Schaefer (1954)^87^ trajectory) and another involving non-linear density-dependence growth with *z* > 1 (i.e., *z* becomes an estimated parameter) which implies that density-dependence effects manifest when the population is near carrying capacity (known in the fishery literature as a Pella–Tomlinson (1969)^37^ surplus-production model). These two basic formulations were combined with the single harvest series and the three alternative fisheries bycatch series to generate a total of six alternative scenarios to describe the population dynamics of the SASL population in northern and central Patagonia.

The logistic model was reparametrized in terms of relative abundance $({P}_{t}=\frac{{N}_{t}}{K})$ to improve the efficiency of the Markov Chain Monte Carlo (MCMC) algorithm used to estimate parameters and to reduce parameter correlations (see 76). Process error (*u*_*t*_ = *e* ^*U*^_*t*_) was accounted for in the state process by assuming independent and multiplicative lognormal error structures^88^. This model also assumed that the pre-harvested population was at the environmental carrying capacity (i.e., *N*_1900–1928_ = *K*). Therefore, the state process was assumed to follow a stochastic transition model as:

4 $$\begin{array}{rcl}{P}_{1900-1928} & = & {e}^{{U}_{1}}\\ {P}_{t} & = & ({P}_{t-1}+{R}_{max}{P}_{t-1}[1-{P}_{t-1}^{z}]-\frac{{H}_{t-1}}{K}-\frac{{M}_{t-1}}{K}){e}^{{U}_{t}}\quad {\rm{for}}\,t > 1928\end{array}$$

where *U*_*t*_ is the total variability in the population growth process in year *t* which was assumed to be a zero mean Gaussian process with variance *σ* ^2^, i.e., *U*_*t*_ ~ *N*(0, *σ* ^2^).

The observation process of the stochastic model assumed that the observed annual numbers of sea lions (*I*_*t*_, *t* = 1972, …, 2013; considering the three correction factors) were noisy measurements that were roughly proportional to the relative abundance (*P*_*t*_)^76^. For process errors, observation error (*v*_*t*_ = *e* ^*V*^_*t*_) was accounted for in the observation process by assuming a multiplicative lognormal error structure. Given the observation errors, the observation equations for each annual time period were:

5 $${I}_{t}=(qK{P}_{t}){e}^{{V}_{t}}$$

where *q* is the detectability coefficient (a proportionality constant representing the fraction of the population observed) and *V*_*t*_ was the extent of the observation error for year *t* and was assumed to have independent and identically distributed normal random variables with variance *τ* ^2^, i.e., *v*_*t*_ ~ *N*(0, *τ* ^2^).

### Parameter estimation

Bayesian estimation in SSMs is a very flexible statistical modelling approach that can handle both process and observation error well. This approach was applied to estimate both the abundance trajectory (*P*_1_, …, *P*_*t*_ with *t* = 1900–2015) and the uncertainty in the parameter estimates. *H*_*t*_ and *M*_*t*_ were considered known in the estimation, and *I*_*t*_ was the observed vector. The unknown parameters in the model were *R*_*max*_, *K*, *z*, *q*, *σ* ^2^ and *τ* ^2^.

Although Bayesian models can include prior knowledge about parameters, here we used vaguely informative prior distributions for the model parameters *K*, *z*, *σ* ^2^ and *τ* ^2^ due to a lack of prior knowledge about their values and distributions for the SASL population. Prior distributions were centred at plausible values and constrained within realistic biological bounds (Table 5). For *K* to be greater than 0 while having a low likelihood of very large values (e.g., 6), we used a lognormal distribution to describe the prior for *K* with a coefficient of variation of 100%. In the case of *z*, we considered a uniform prior distribution over the interval 0.0001 to 10. This ensures a fair non-informative prior for the shape parameter. Priors for the process error variance *p*(*σ* ^2^) and observation error variance *p*(*τ* ^2^) were chosen to be diffuse inverse-gamma distributions. The choice of this distribution implied that the parameters are approximately uniform on ln(x) (Jeffrey’s prior) and have the property that lower weights are assigned to higher values of *σ* ^2^ and *τ* ^2^, which helps to prevent implausibly large posterior values of *σ* ^2^ and *τ* ^2^ ^29^. As a result, inferences based on the gamma assumption were scale invariant and would not be affected by changing the scale of the variance parameter.

**Table 5:** Estimable parameters and prior specifications for Bayesian state-space models.

Parameter	Description	Default prior	Alternative prior
*z*	shape parameter	*z* ~ *unif*(0.0001, 10)	*z* ~ *unif*(0.0001, 20) (sen1)
R_max_	maximum rate of increase	R_max_ ~ lnorm(μ_Rmax_ = −2.9, σ_Rmax_ = 0.5)	
*K*	carrying capacity	*K* ~ *lnorm*(*μ*_*K*_ = 5.7, *σ*_*K*_ = 0.8)	*K* ~ *unif*(0.0001, 1000) (sen2) *K* ~ *lnorm*(*μ*_*K*_ = 7.4, *σ*_*K*_ = 0.8) (sen3) *K* ~ *lnorm*(*μ*_*K*_ = 4, *σ*_*K*_ = 0.8) (sen4) *K* ~ *lnorm*(*μ*_*K*_ = 5.7, *σ*_*K*_ = 1.25) (sen5)
*q*	detectability coefficient	*q* ~ *beta*(2.2, 2)	
*σ* ^2^	process variance	*σ* ^2^ ~ *invgamma*(1, 0.4)	*σ* ^2^ ~ *invgamma*(2, 0.8) (sen6) *σ* ^2^ ~ *invgamma*(0.5, 0.2) (sen7)
*τ* ^2^	observation variance	*τ* ^2^ ~ *invgamma*(3, 0.8)	*τ* ^2^ ~ *invgamma*(6, 1.6) (sen8) *τ* ^2^ ~ *invgamma*(1.5, 0.4) (sen9)

Alternative prior specifications were considered in the sensitivity analyses (sen1–9).

To specify a prior distribution for *R*_*max*_, we used the best guess estimates for this parameter from a deterministic surplus production model (maximum likelihood estimate) developed for the colonies of north Patagonia^23^ and other analyses of SASL population trends for the Southwestern Atlantic Ocean^8, 17, 22, 25, 40, 89^. Estimates for *R*_*max*_ were selected from models that had the strongest statistical fits and were judged to be biologically relevant. The different estimates suggest that mean values for *R*_*max*_ range from 0.037 to 0.084. Due to uncertainty about an appropriate prior mean for *R*_*max*_, we choose a vague lognormal prior for *R*_*max*_ with a prior mean *μ*_*Rmax*_ = 0.055 and CV of 50%, allowing sufficient flexibility to estimate the probable value of *R*_*max*_ given the observed data (Table 5).

The prior distribution for *q* was based on previous independent estimates developed to account for the proportion of animals that are missed during surveys due to imperfect detection (i.e., animals at sea) and/or sampling variation^23, 52^. The different factors considered available data from life tables, mark-recapture or satellite-tracked studies to correct survey data using the percentage of the different age-sex classes present on land. The mean values varied between 0.38 and 0.74. Here, a beta distribution was assigned to *q*, with large bounds and a mean set equal to *μ*_*q*_ = 0.52 (Table 5). A beta distribution for *q* is particularly convenient because this distribution falls between 0 and 1 and can take on a wide variety of shapes.

For parameter estimates, their full posterior distributions and states for the SSM were approximated using Markov Chain Monte Carlo (MCMC) techniques. All the Bayesian estimations were performed using the WinBUGS software^90, 91^ (available at http://www.mrc-bsu.cam.ac.uk/software/bugs/the-bugs-project-winbugs/) and implemented with the R programming language^92^ through the R2WinBUGS package^93^. WinBUGS uses the Gibbs sampling algorithm to sample from the posterior distributions of parameters. The WinBUGS code is shown in Appendix A. In all MCMC simulations, three independent chains were run for 1,000,000 iterations. From each chain, the first 100,000 iterations were discarded to remove any dependence of the MCMC samples on the initial conditions. After this burn-in phase, a thinning rate of 50 was used (i.e., only every 50^th^ iteration was kept to reduce the MCMC sampling autocorrelation^6^.

The convergence diagnostics of the MCMC draws onto stable estimates were checked using the CODA package for R (Convergence Diagnostics and Output Analysis^94, 95^), adopting minimal thresholds of *p* = 0.05 for Geweke’s diagnostic^96^, the two-stage Heidelberger-Welch stationary test^97^, and the Gelman-Rubin diagnostic ($\hat{R}=1.05$ for all variables)^98, 99^. We also monitored the trace plots of parameter estimates over the MCMC iterations to understand the behaviour of the chain and diagnosed the autocorrelation plot for key parameters. Bayesian credibility intervals for all parameters were estimated by calculating the 2.5^th^ and 97.5^th^ percentiles of the posterior distributions generated in WinBUGS.

Posterior distributions may be highly influenced by the shape of the prior and by priors centred on inaccurate values, especially when the data are not very informative^29^. Hence, model runs with different prior distributions were conducted to test the sensitivity of the parameter estimates to the base-case set of prior probability specifications. We focused on parameters with vague prior specifications due to the lack of prior knowledge. The sensitivity of the results to the prior distributions was tested by changing the distribution of one parameter while keeping the others on the base-case set. These tests included (Table 5):

- Specifying a less informative prior for *z*, i.e., a uniform prior distribution over the interval 0.0001 to 20.
- To analyse the sensitivity to *K*, three tests were done: (a) specifying the prior for *K* as uniform rather than lognormal; (b) using priors that were positively or negatively biased from the base-case. The probability distribution of *K* was assumed to have had means on the log scale that were 30% higher and lower than the base-case configuration. In contrast, the prior CVs were held constant; (c) specifying a prior that was even vaguer than the base-case. The lognormal distribution was used for *K* with a coefficient of variation of 200%.
- Specifying the inverse-gamma distributions on σ^2^ and τ^2^ to half/double the baseline canonical parameter.

The sensitivity of the results to the datasets used to fit the model was also tested. First, all models were fitted only to abundance and annual harvest estimates. Then, estimates for bycatch history were added in new runs. In those runs, the model priors were the same as in the previous models.

The goodness-of-fit of the competing surplus production models was inferred by the Deviance Information Criterion (*DIC* ^100^). Model residuals were also used to measure the goodness-of-fit of the alternative production models. Non-random patterns in the residuals indicated that the observable vector (*I*_*t*_) did not conform to one or more of the model assumptions.

The consistency between the model and the data was checked using Bayesian posterior predictive checking procedures^101^ designed to check the ability of the model *a posteriori* to replicate abundance data similar to those observed. We calculated the Bayesian *p*-value, which is the probability that the “ideal” data could be as extreme as or more extreme than the observed data^101^. We assumed a reasonable fit if 0.1< *p*-value < 0.9. To obtain the “ideal” datasets, replicate datasets were assembled using the same models that were fit to the actual dataset at each MCMC iteration using the current parameter values.

## Electronic supplementary material

Supplementary Information
