# Supplementary material for: Analysing the natural population growth of a large marine mammal after a depletive harvest
Source: Sci Rep. 2017 Jul 13;7:5271. doi: 10.1038/s41598-017-05577-6 (PMC5509669; doi:10.1038/s41598-017-05577-6)
Supplement: Supplementary file 1 — Supplementary Information [file 41598_2017_5577_MOESM1_ESM.doc]

**Analysing the natural population growth of a large marine mammal after a depletive harvest**

Romero M.A., Grandi M.F., Koen-Alonso M., Svendsen G., Ocampo Reinaldo M., García N.A., Dans S.L., González R. and Crespo E.A.

**Supplementary tables**

Table S1. Abundance, harvest and bycatch series (number of individuals) used in this study.
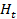
: annual harvests values;
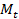
: annual bycatch values (TC: Total Catch set; AC: Average Catch set; MC: Maximum Catch set);
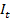
: observed annual numbers of sea lions (corrected by the three Correction Factors, Table 4).

| Year | 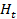 |  |  | 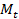 |  |  | 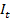 |
| --- | --- | --- | --- | --- | --- | --- | --- |
|  |  |  | TC | AC | MC |  |  |
| 1929 | 7 |  |  |  |  |  |  |
| 1930 | 54 |  |  |  |  |  |  |
| 1931 | 275 |  |  |  |  |  |  |
| 1932 | 956 |  |  |  |  |  |  |
| 1933 | 2455 |  |  |  |  |  |  |
| 1934 | 4948 |  |  |  |  |  |  |
| 1935 | 8221 |  |  |  |  |  |  |
| 1936 | 11713 |  |  |  |  |  |  |
| 1937 | 14769 |  |  |  |  |  |  |
| 1938 | 16900 |  |  |  |  |  |  |
| 1939 | 17907 |  |  |  |  |  |  |
| 1940 | 17850 |  |  |  |  |  |  |
| 1941 | 16953 |  |  |  |  |  |  |
| 1942 | 15497 |  |  |  |  |  |  |
| 1943 | 13743 |  |  |  |  |  |  |
| 1944 | 11898 |  |  |  |  |  |  |
| 1945 | 10108 |  |  |  |  |  |  |
| 1946 | 8458 |  |  |  |  |  |  |
| 1947 | 6995 |  |  |  |  |  |  |
| 1948 | 5730 |  |  |  |  |  |  |
| 1949 | 4659 |  |  |  |  |  |  |
| 1950 | 3767 |  |  |  |  |  |  |
| 1951 | 3031 |  |  |  |  |  |  |
| 1952 | 2430 |  |  |  |  |  |  |
| 1953 | 1942 |  |  |  |  |  |  |
| 1954 | 1549 |  |  |  |  |  |  |
| 1955 | 1233 |  |  |  |  |  |  |
| 1956 | 980 |  |  |  |  |  |  |
| 1957 | 778 |  |  |  |  |  |  |
| 1958 | 617 |  |  |  |  |  |  |
| 1959 | 489 |  |  |  |  |  |  |
| 1960 | 388 |  |  |  |  |  |  |
| 1972 |  |  |  |  |  |  | 8657 |
| 1973 |  |  |  |  |  |  | 10960 |
| 1974 |  |  |  |  |  |  | 11806 |
| 1975 |  |  |  |  |  |  | 15704 |
| 1981 |  |  |  |  |  |  | 16869 |
| 1982 |  |  |  |  |  |  | 14172 |
| 1983 |  |  |  |  |  |  | 15374 |
| 1984 |  |  |  |  |  |  | 13428 |
| 1985 |  |  |  |  |  |  | 16631 |
| 1989 |  |  | 232 | 253 | 859 |  | 21590 |
| 1990 |  |  | 241 | 263 | 899 |  | 21608 |
| 1991 |  |  | 216 | 236 | 801 |  |  |
| 1992 |  |  | 232 | 255 | 864 |  |  |
| 1993 |  |  | 269 | 297 | 1023 |  |  |
| 1994 |  |  | 283 | 314 | 1082 |  | 25279 |
| 1995 |  |  | 306 | 341 | 1202 |  | 27557 |
| 1996 |  |  | 354 | 394 | 1399 |  | 36703 |
| 1997 |  |  | 357 | 400 | 1432 |  | 36135 |
| 1998 |  |  | 352 | 393 | 1403 |  | 37391 |
| 1999 |  |  | 406 | 448 | 1584 |  | 41558 |
| 2000 |  |  | 338 | 368 | 1285 |  | 39656 |
| 2001 |  |  | 444 | 484 | 1696 |  | 50597 |
| 2002 |  |  | 394 | 432 | 1519 |  |  |
| 2003 |  |  | 418 | 456 | 1598 |  |  |
| 2004 |  |  | 443 | 483 | 1703 |  |  |
| 2005 |  |  | 275 | 302 | 1074 |  | 55370 |
| 2006 |  |  | 407 | 443 | 1573 |  | 58958 |
| 2007 |  |  | 392 | 427 | 1510 |  | 58307 |
| 2008 |  |  | 323 | 353 | 1248 |  |  |
| 2009 |  |  | 258 | 282 | 999 |  | 64850 |
| 2010 |  |  | 302 | 330 | 1170 |  |  |
| 2011 |  |  | 312 | 340 | 1209 |  |  |
| 2012 |  |  | 237 | 260 | 921 |  |  |
| 2013 |  |  | 234 | 256 | 906 |  |  |

Table S2. Correlation coefficients between the parameters for the six Bayesian state-space surplus production models (linear and non-linear density-dependence models under the three bycatch indices). TC, AC, and MC respectively denote Total Catch, Average Catch and Maximum Catch set estimated to reconstruct bycatch history.

| Linear density-dependence |  |  |  |  |  |  |
| --- | --- | --- | --- | --- | --- | --- |
| TC |  | 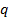 | 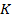 | 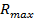 | 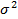 |  |
|  | 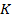 | -0.187 |  |  |  |  |
|  | 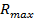 | -0.012 | -0.308 |  |  |  |
|  | 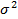 | -0.024 | 0.041 | 0.023 |  |  |
|  | 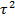 | -0.017 | 0.019 | 0.004 | 0.193 |  |
| AC |  |  |  |  |  |  |
|  | 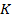 | -0.158 |  |  |  |  |
|  | 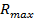 | -0.019 | -0.300 |  |  |  |
|  | 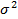 | -0.029 | 0.047 | 0.032 |  |  |
|  | 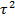 | -0.010 | 0.012 | 0.009 | 0.182 |  |
| MC |  |  |  |  |  |  |
|  | 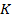 | -0.207 |  |  |  |  |
|  | 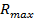 | 0.021 | -0.305 |  |  |  |
|  | 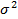 | -0.014 | 0.041 | 0.009 |  |  |
|  | 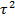 | -0.016 | 0.002 | 0.017 | 0.190 |  |
|  |  |  |  |  |  |  |
| Non-linear density-dependence | |  |  |  |  |  |
| TC |  | 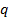 | 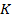 | 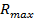 | 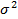 | 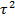 |
|  | 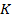 | -0.184 |  |  |  |  |
|  | 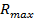 | -0.018 | -0.172 |  |  |  |
|  | 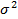 | -0.014 | 0.012 | 0.016 |  |  |
|  | 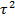 | -0.008 | 0.004 | 0.005 | 0.175 |  |
|  | 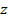 | 0.065 | 0.215 | -0.105 | -0.055 | -0.017 |
| AC |  |  |  |  |  |  |
|  | 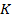 | -0.174 |  |  |  |  |
|  | 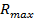 | -0.022 | -0.158 |  |  |  |
|  | 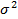 | -0.016 | 0.024 | 0.012 |  |  |
|  | 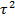 | -0.003 | -0.002 | 0.004 | 0.188 |  |
|  | 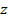 | 0.066 | 0.211 | -0.094 | -0.055 | -0.016 |
| MC |  |  |  |  |  |  |
|  | 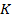 | -0.197 |  |  |  |  |
|  | 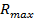 | 0.008 | -0.175 |  |  |  |
|  | 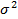 | -0.008 | -0.004 | -0.008 |  |  |
|  | 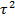 | -0.001 | 0.003 | -0.007 | 0.184 |  |
|  | 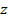 | 0.059 | 0.208 | -0.093 | -0.075 | -0.022 |

Table S3. Fishing effort time-series (from the Fisheries and Aquaculture National Department of Argentina) of the trawl fleet which operates in northern and central Patagonia. Nominal fishing effort (
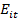
, number of fishing days per year) is listed according to fishing types considered to reconstruct the South American sea lion bycatch history (see full fishing type description from 75,76).

|  | Fishing Type | | | | | | |
| --- | --- | --- | --- | --- | --- | --- | --- |
| Year | 2 | 3 | 4 | 5 | 6 | 7 | 8 |
| 1989 | 19710 |  | 5114 | 281 | 1815 | 1480 | 96 |
| 1990 | 20148 | 80 | 4753 | 310 | 2570 | 1749 | 96 |
| 1991 | 17253 | 404 | 4341 | 502 | 3124 | 1561 | 96 |
| 1992 | 18241 | 837 | 5150 | 853 | 3475 | 1350 | 96 |
| 1993 | 20867 | 558 | 7518 | 1026 | 4447 | 1562 | 96 |
| 1994 | 22051 | 406 | 9436 | 1419 | 4288 | 1454 | 60 |
| 1995 | 24171 | 428 | 11167 | 1370 | 5186 | 120 | 30 |
| 1996 | 28449 | 459 | 11744 | 772 | 6289 | 0 | 0 |
| 1997 | 27994 | 449 | 13728 | 480 | 7039 | 0 | 0 |
| 1998 | 28060 | 374 | 13424 | 643 | 6012 | 0 | 0 |
| 1999 | 34183 | 566 | 11450 | 821 | 5003 | 0 | 0 |
| 2000 | 30092 | 446 | 7905 | 1332 | 1397 | 0 | 0 |
| 2001 | 39617 | 537 | 9360 | 1745 | 2396 | 0 | 0 |
| 2002 | 33770 | 594 | 9988 | 1861 | 3665 | 0 | 0 |
| 2003 | 36849 | 525 | 8798 | 2997 | 2588 | 0 | 0 |
| 2004 | 39067 | 495 | 8552 | 3255 | 3150 | 0 | 0 |
| 2005 | 23558 | 258 | 6109 | 1699 | 3167 | 0 | 0 |
| 2006 | 35648 | 320 | 7601 | 3011 | 3601 | 0 | 0 |
| 2007 | 34278 | 392 | 7405 | 3910 | 3124 | 0 | 0 |
| 2008 | 27497 | 375 | 7465 | 4177 | 2921 | 0 | 0 |
| 2009 | 21529 | 371 | 6043 | 4435 | 2641 | 0 | 0 |
| 2010 | 25548 | 377 | 6490 | 4367 | 3058 | 0 | 0 |
| 2011 | 26284 | 420 | 6702 | 4440 | 3312 | 0 | 0 |
| 2012 | 19600 | 394 | 5640 | 3699 | 2789 | 0 | 0 |
| 2013 | 19544 | 354 | 5451 | 4095 | 2319 | 0 | 0 |

Type 2. Used during diurnal bottom trawling by Distal Costal (DC) vessels. The mouth of the net is 3 m high and 25 m wide, and the target species is hake.

Type 3. Used during diurnal bottom trawling by Freezing (FZ) and Factory (F) vessels. The mouth of the net is 3 m high and 42 m wide, and the target species is shrimp.

Type 4. Used during diurnal bottom trawling by F vessels. The mouth of the net is 3.5 m high and 49 m wide, and the target species are hake, grouper, Brazilian sandperch, pink cusk-eel and squid.

Type 5. Used during diurnal bottom trawling by two-rig system FZ vessels. Each net has a mouth 0.8 to 1.5 m high and 28 m wide, and the target species is shrimp.

Type 6. Used during diurnal bottom trawling by F vessels. The mouth of the net is 5 m high and 42 m wide, and the target species are hake, grouper, Brazilian sandperch, pink cusk-eel and squid.

Type 7. Used during diurnal bottom trawling by factory vessels. The mouth of the net is 7 m high and 52 m wide, and the target species is shrimp.

Type 8. Used during nocturnal mid-water trawling by factory vessels. The mouth of the net is 20 m high and 40 m wide, and the target species is shrimp.

**Supplementary figures**


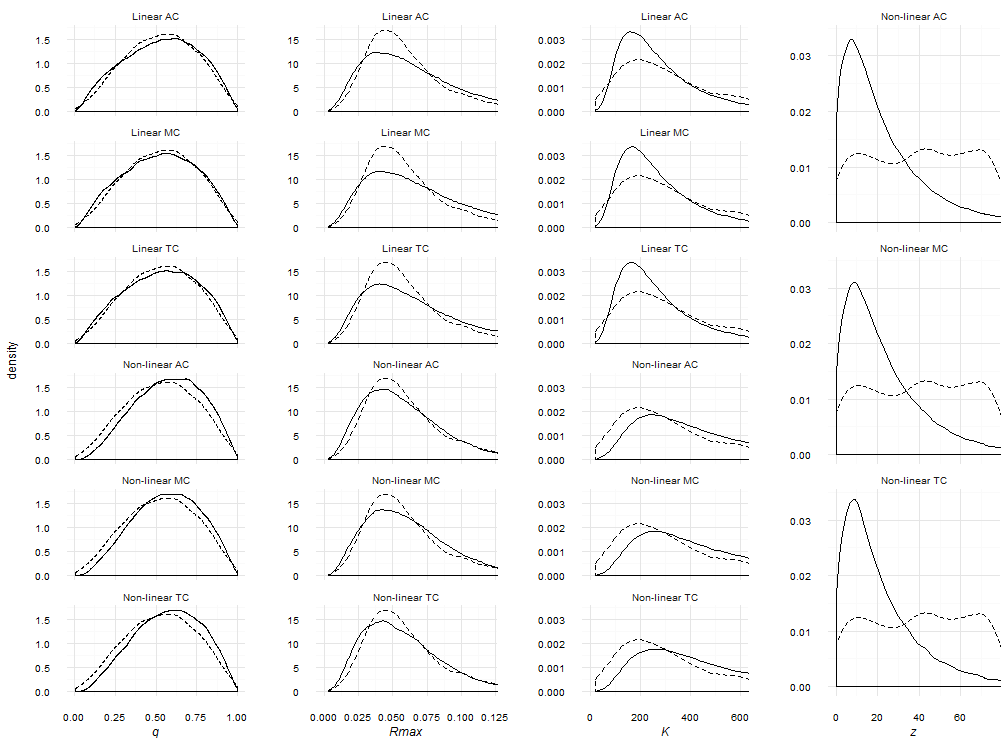


Figure S1. Prior (dashed line) and posterior (solid line) probability density distributions of the parameters (detectability coefficient
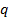
, carrying capacity
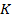
, maximum rate of increase
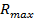
, shape parameter
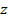
) from the Bayesian state-space surplus production models (linear and non-linear density-dependence models according to the three bycatch estimated series). TC: Total Catch set; AC: Average Catch set; MC: Maximum Catch set.


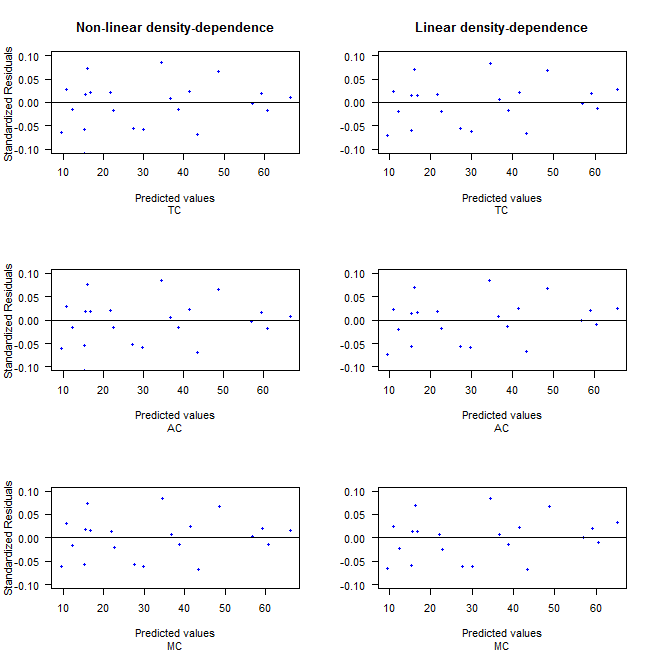


Figure S2. Standardized residuals of observed annual number of South American sea lion *Otaria flavescens* from the six Bayesian state-space surplus production models (linear and non-linear density-dependence models according to the three bycatch estimated series) plotted against predicted values. TC: Total Catch set; AC: Average Catch set; MC: Maximum Catch set.

**Appendix A.** Bayesian non-linear surplus-production state-space model in WinBUGS with both process and observation error and Harvest (
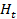
) and bycatch series (
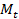
).

#### using Millar and Meyer (2000) parameterization of a non-linear surplus production model ####

model {#### unobserved state variable P = production =
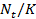
, assuming
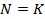
, from
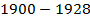
 ####

#### Process´s error ######

for (j in 1:29) {

Pmean[j] <- 0

P[j] ~ dlnorm(Pmean[j],sigma2)

}

For (i in 30:N) {

Pmean[i] <- log(max(P[i-1] + r*P[i-1]*(1-pow(P[i-1],z))-K*H[i-1]-K*M[i-1],0.01))

P[i] ~ dlnorm(Pmean[i],sigma2)

}

**####** Observation´s error **####**

for (i in 1:N) {

Imean[i] <- log(P[i]*X)

I[i] ~ dlnorm(Imean[i],tau2)

#### Prior on z (shape parameter) ####

tita ~ dunif(0.0001,10)

#### Prior on r ####

rl ~ dlnorm(-2.9,2) #original

#### Prior on
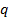
 (detectability coefficient) ####


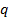
 ~dbeta(2.2,2)

X <-
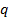
*k

#### Prior on
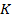
 ####

k ~ dlnorm(5.68,1.25)

K<-1/k

#### Priors on sigma2 and tau2 ####

a1<-1; b1<-0.4

sigma2 ~ dgamma(a1,b1)

Sigma2<-1/sigma2;

c1<-3; d1<-0.8

tau2 ~ dgamma(c1,d1)

Tau2<-1/tau2

#### Derived quantities ####

for (i in 1:N) {

predictedI[i]<-exp(Imean[i]) # estimated observed variable

predictedN[i]<-exp(Imean[i])/q #estimated unobserved state variable

}

#### Prediction: M-year extension to process equation ####

for(i in (N+1):(N+5)) {

Pmean[i] <- log(max(P[i-1] + r*P[i-1]*(1-pow(P[i-1],tita)),0.01))

P[i] ~ dlnorm(Pmean[i],sigma2)

Imean[i] <- log(P[i]*X)

predictedI.new[i]<-exp(Imean[i])

predictedN.new[i]<-exp(Imean[i])/q

}

}

#### input data file and initial conditions ####
